# Fine Particulate Air Pollution and First Hospital Admissions for Ischemic Stroke in Beijing, China

**DOI:** 10.1038/s41598-017-04312-5

**Published:** 2017-06-20

**Authors:** Yaohua Tian, Xiao Xiang, Yiqun Wu, Yaying Cao, Jing Song, Kexin Sun, Hui Liu, Yonghua Hu

**Affiliations:** 1 Department of Epidemiology and Biostatistics, School of Public Health, Peking University, No. 38 Xueyuan Road, 100191 Beijing, China; 2 Medical Informatics Center, Peking University, No. 38 Xueyuan Road, 100191 Beijing, China

## Abstract

The primary objective of this study was to assess the association between short-term changes in ambient fine particulate matter (PM_2.5_) and first hospital admissions for ischemic stroke. We identified 63,956 first hospital admissions for ischemic stroke from the Beijing Medical Claim Data for Employees from January 1, 2010, through June 30, 2012. A generalized additive Poisson model was applied to explore the association between PM_2.5_ and admissions for ischemic stroke. We also explore the effect modification of risk by age and gender. The exposure–response relationship between PM_2.5_ and admissions for ischemic stroke was approximately linear, with a relatively stable response at lower concentrations (<100 μg/m^3^) and a steeper response at higher concentrations. A 10 μg/m^3^ increase in the same-day PM_2.5_ concentration was associated with 0.31% (95% CI, 0.17–0.45%, *P* < 1.57 × 10^−5^) increase in the daily admissions for ischemic stroke. The association was also statistically significant at lag 1, 2, 3, 0–2 and 0–4 days. Subgroup analyses showed that the association was not different between patients ≥65 years and <65 years old or between males and females. In conclusion, short-term exposure to PM_2.5_ was positively associated with first hospital admissions for ischemic stroke in Beijing, China.

## Introduction

Stroke is the second most common cause of death and the third leading cause of disability-adjusted life years globally^1, 2^. In 2010, approximately 16.9 million new cases of stroke were diagnosed and 5.9 million stroke-related deaths occurred worldwide^3^. In China, stroke is the leading cause of death and adult disability, with an estimated 2.5 million new stroke cases and 1.6 million deaths from stroke occurring each year. In line with other countries, ischemic stroke is the predominant subtype of stroke in China, accounting for 43–79% of all stroke events^4^. Despite major improvements in primary and secondary prevention for stroke over the last decades, the global incidence of stroke continues to increase, particularly in economically transitioning countries including China due to the rapid growth and aging of population as well as changes in environmental and lifestyle factors^3^. Additionally, stroke is responsible for considerable patient suffering and imposes a substantial economic burden to society^4, 5^. Therefore, it is of great significance to identify modifiable risk factors for stroke from public health perspective.

A mounting body of evidence suggests that air pollution is an important risk factor for cardiovascular morbidity and mortality^6–8^, accounting for over three million deaths worldwide annually^9^. With the rapid industrialization and economic growth in the past several decades, air pollution has become an increasingly serious concern in China. Haze-fog pollution, an atmospheric phenomenon caused by the presence of dust, smoke and other dry particles at high concentration, has frequently occurred in China in recent years^10^. From 2013 to 2014, the annual mean concentrations of ambient fine particulate matter (PM_2.5_, particles with an aerodynamic diameter ≤2.5 μm) for all of the 31 provincial capital cities in China failed to reach the Chinese Ambient Air Quality Standards (CAAQS) Grade I standard (15 μg/m^3^), and only three cities met the CAAQS Grade II standards (35 μg/m^3^). Beijing, the capital of China, was experiencing more serious haze-fog pollutions because of coal burning and adverse weather conditions as well as the automobile exhaust emitted by the rapidly increasing transportation vehicles^11, 12^. The severe haze-fog pollution has attracted extensive public concern due to the adverse health effects.

A meta-analysis that combined the results from prior studies using mortality and/or admission data indicated that various gaseous pollutants including carbon monoxide, sulphur dioxide, nitrogen dioxide, and PM_10_ (particles with an aerodynamic diameter ≤10 μm) were significantly associated with increased risk of ischemic stroke^13^. However, only a mere handful of studies have been undertaken which specifically evaluated the health effects of PM_2.5_ on ischemic stroke, due primarily to the lack of PM_2.5_ monitoring data. Researchers have suggested that PM_2.5_ was more harmful to human health than PM_10_, because PM_2.5_ can penetrate more deeply into the lung and carry larger concentrations of adsorbed or condensed toxic air pollutants per unit mass with its greater surface area^14, 15^. In addition, previous studies were primarily conducted in Western developed countries; however, because of the considerable differences in the level of pollution, weather patterns, and population susceptibility, the association between PM_2.5_ and ischemic stroke is still unclear in developing countries.

The primary interest outcome in prior studies that evaluated the short-term effects of PM_2.5_ on ischemic stroke hospitalizations was the daily total number of admissions regardless of first or repeated admissions, and all admissions were treated as independent events. A meta-analysis reported that 1-year and 5-year stroke recurrence rates were 11.1% and 26.4%, respectively^16^. Thus, admissions for recurrent stroke may account for a considerable proportion of daily hospital admissions for stroke. While first-ever and recurrent stroke share similar risk factors and clinical manifestations, their clinical characteristics and pathogenetic features are not exactly the same^17, 18^. Furthermore, several studies indicated that a history of the disease had considerable influence on the association between air pollution and the specific health outcome^7, 19^. On the other hand, repeated admissions could cause a temporal dependence among the hospitalization counts, leading to an underestimation of the variance of air pollution risk estimates^20^. Based on these findings, it seems plausible that air pollution may have a differential effect on first-ever vs. recurrent stroke events. Therefore, studies that specifically examine the association between PM_2.5_ and first-ever stroke are needed to better understand the real impact of ambient fine particulate matter on public health.

The primary objective of this study was to examine the short-term effect of PM_2.5_ on first admissions for ischemic stroke in Beijing, China.

## Methods

### Data collection

Data on daily admissions for ischemic stroke from January 1, 2010, through June 30, 2012, was obtained from Beijing Medical Claim Data for Employees (BMCDE). Medical claim data for all working or retired employees who are covered by basic medical insurance in Beijing were recorded in BMCDE. The medical information recorded in BMCDE includes basic demographics (age and sex), admission date, medication use, clinical diagnosis in Chinese and corresponding International Classification of Diseases, 10th Revision (ICD-10) codes, and reimbursement information. The information on brain computed tomography or magnetic resonance imaging use was also recorded in the database. Hospital admissions for ischemic stroke were identified according to the principal diagnosis, using the ICD-10 code of I63. Specifically, subjects who had been hospitalized for stroke (I60–I64) during the five years preceding the index event were excluded, and we focused on the first admissions for ischemic stroke in this study. We also used the corresponding Chinese diagnoses to check the identified cases, ensuring that only the first admissions could be included in this study.

The air pollution data, hourly PM_2.5_ concentration from January 1, 2010, through June 30, 2012, was obtained from a web platform (http://www.stateair.net/web/historical/1/1.html) run by the US embassy, which established an ambient air quality monitoring station on the rooftop of embassy building located in Chaoyang district, Beijing. A prior study has indicated that the PM_2.5_ concentrations obtained from the monitoring station exhibited approximately the same trend as citywide PM_2.5_ observations^21^. Furthermore, 79.2% of Beijing’s total population resided within a 40-km radius of the U.S. embassy ambient monitoring station. All areas of high population density (>5000 people/km^2^), 97.8% (44/45) of the tertiary hospitals and 79.3% (69/87) of the secondary hospitals in Beijing that admit ischemic stroke cases located within a 40-km radius of the monitoring station. It has been suggested that the monitoring data could be used as a good proxy for personal exposure among individuals residing <40 km from the monitoring station^8, 14^. In addition, only urban residents were included in this study to further reduce exposure misclassification. To allow for the effects of weather conditions, meteorological data on daily 24-hour average temperature (°C) and relative humidity (%) was obtained from the Chinese Meteorological Bureau over the same period.

### Statistical Analysis

Daily average PM_2.5_ concentration, daily admissions for ischemic stroke, and weather conditions were linked by date and, thus could be analyzed with a time-series design. Because daily hospital admission for ischemic stroke was rare, a generalized additive Poisson model was applied to explore the association between PM_2.5_ and ischemic stroke, after adjusting for day of week (DOW), calendar time, public holiday, and daily average temperature and relative humidity. The penalized spline (*ps*) function of calendar time with 10 degrees of freedom (*df*) was used to adjust for seasonality and long-term trends^8^. We also used the *ps* functions of daily average temperature (*df* = 3) and relative humidity (*df* = 3) to allow for the potential nonlinear confounding effects of weather conditions^22, 23^. The DOW and public holiday were also incorporated in the model to control for the difference in the baseline hospital admission rates for each day. After the basic model was established, the variable of PM_2.5_ concentration was introduced. The final model was described below:

$$\begin{array}{c}\mathrm{Log}[{\rm{E}}({{\rm{Y}}}_{{\rm{t}}})]={\rm{\alpha }}+{{\rm{\beta }}}_{1}{\rm{PM}}{2.5}_{{\rm{t}}-{\rm{i}}}+{{\rm{\beta }}}_{2}{\rm{DOW}}+{{\rm{\beta }}}_{3}({\rm{public}}\,{\rm{holiday}})\\ +\,ps({\rm{calendar}}\,{\rm{time}},df=10)+ps({{\rm{Temp}}}_{0},df=3)+ps({{\rm{RH}}}_{0},df=3)\end{array}$$

where, E(Y_t_) is the expected number of admissions for ischemic stroke on day t; α is the model intercept; PM2.5_t−i_ is the mean PM_2.5_ concentration on day t, and i is the day lag; β represents regression coefficient; DOW is the day of the week; public holiday is a binary variable indicating a public holiday on day t (coded as 0 indicates no holiday, and 1 indicates a holiday); *ps*() indicates penalized spline function; Temp_0_ and RH_0_ indicate the daily mean temperature and relative humidity on the current day, respectively. The results are presented as the percentage change and 95% confidence interval (CI) in the daily ischemic stroke admissions per 10 μg/m^3^ increase in PM_2.5_ concentration. Smoothing function was used to graphically analyze the exposure-response relationship between the log-relative risk of ischemic stroke and PM_2.5_ concentration.

To examine the temporal association of PM_2.5_ concentration with ischemic stroke admission, we fitted the models with different lag structures from the current day (lag0) up to 4 lag days (lag4). Considering that single-day lag models may underestimate the effect of pollutant^24^, we also evaluated the cumulative effects using 3-day (lag0–2) and 5-day (lag0–4) moving averages of PM_2.5_ concentrations.

Stratified analyses were conducted to examine whether the association differed by age (≥65 years and <65 years) and gender. The Z-test was applied to test the statistical significance of differences by age or gender^25^. We also conducted sensitivity analyses to examine the robustness of the results in terms of the *df* in the smooth function of time trend (8–12), daily mean temperature (2–6) and daily relative humidity (2–6).

All statistical analyses were carried out using R Programming Language (V.3.2.2, R Development Core Team) with the “*mgcv*” and “*nlme*” packages. All statistical tests were two-sided, and tests of statistical significance were set at *P* < 0.05.

## Results

Table 1 summarizes the basic characteristics for our study. A total of 63,956 first hospital admissions for ischemic stroke were identified from BMCDE database between January 1, 2010 and June 30, 2012. There were 66.5% male patients, and 56.7% patients were ≥65 years old. The mean (standard deviation, SD) age of the ischemic stroke patients was 66.4 (12.1) years.

**Table 1:** Demographic characteristics of Ischemic Stroke Admissions in Beijing, China, January 1, 2010 to June 30, 2012.

Variable	No.
Total	63,956
Gender	
Male (%)	42,529 (66.5)
Female (%)	21,427 (33.5)
Age (year) (mean ± SD)	66.4 ± 12.1
<65 (%)	27,706 (43.3)
≥65 (%)	36,250 (56.7)

The summary statistics for daily ischemic stroke admissions, air pollution and weather conditions from January 1, 2010 to June 30, 2012 are shown in Table 2. The mean daily count for ischemic stroke admission was 70 during the study period. The overall mean daily PM_2.5_ concentration was 99.5 μg/m^3^, with a range from 7.2 to 492.8 μg/m^3^. According to the CAAQS Grade II standards for daily mean concentrations of PM_2.5_ (75 μg/m^3^), 45.4% (414 days) of the daily PM_2.5_ concentrations were up to the standard. However, in terms of the WHO Air Quality Guidelines for 24-hour average PM_2.5_ concentration (25 μg/m^3^), only 13.6% (124 days) days met the standard. The means (SD) of temperature and relative humidity were 12.6 °C (11.6 °C) and 48.6% (20.3%), respectively.

**Table 2:** Summary statistics for daily number of hospital admissions for ischemic stroke, daily fine particulate matter (PM_2.5_) concentrations and weather conditions in Beijing, China, January 1, 2010 to June 30, 2012.

Variable	Mean ± SD	Minimum	Percentile			Maximum
			25th	50th	75th	
Daily admission	70.1 ± 43.8	1	12	86	102	167
PM_2.5_ (μg/m^3^)	99.5 ± 75.3	7.2	42.5	82.8	133.3	492.8
Temperature(°C)	12.6 ± 11.6	−12.5	1.5	14.1	23.8	34.5
Relative humidity (%)	48.6 ± 20.3	9	30	48	66	92

**Figure 1:**
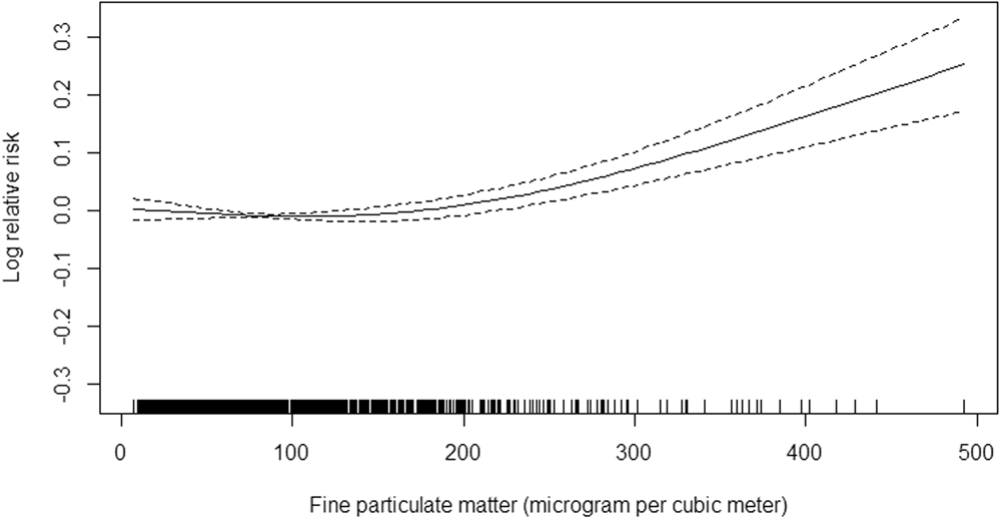
The smoothed exposure-response curves of daily average PM_2.5_ concentrations at current-day against ischemic stroke hospital admission. Note: The X-axis is the current-day (lag 0 day) fine particulate matter (PM_2.5_) concentrations (mg/m^3^). Y-axis is the predicted log (relative risk (RR), after adjusting for calendar time, day of the week, public holiday, current-day temperature, and relative humidity, is shown by the solid line, and the dotted lines represent the 95% CI.

There was a clear dose–response relationship between PM_2.5_ concentration and the count of daily admissions for ischemic stroke (Fig. 1). The relationship was approximately linear, with a tiny fluctuation at lower concentrations (<100 μg/m^3^) and a sharper response at higher concentrations.

Table 3 shows the association between PM_2.5_ concentration and ischemic stroke admissions for different lag structures. We observed significant associations between ischemic stroke admissions and PM_2.5_ concentrations on the current day (lag0) and lag 1, 2 and 3 days after adjustment for calendar time, day of the week, public holiday, and weather conditions. A 10 μg/m^3^ increase in PM_2.5_ concentration at lag 0, 1, 2 and 3 days corresponded to a 0.31% (95% CI, 0.17–0.45%), 0.48% (95% CI, 0.35–0.60%), 0.48% (95% CI, 0.37–0.58%), and 0.32% (95% CI, 0.22–0.43%) increase in ischemic stroke admissions, respectively. An increment of 10 μg/m^3^ in 3-day (percentage change, 0.80%; 95% CI, 0.64–0.96%) and 5-day (percentage change, 0.75%; 95% CI, 0.58–0.93%) average PM_2.5_ concentrations were also significantly associated with increased ischemic stroke admissions.

**Table 3:** Percentage change with 95% CI in ischemic stroke admissions associated with a 10 μg/m^3^ increase in fine particulate matter (PM_2.5_) concentration for different lag structures.

Lag days	Percentage change	95% CI	*P*
Lag 0 days	0.31	0.17–0.45	1.57e-05
Lag 1 days	0.48	0.35–0.60	1.35e-14
Lag 2 days	0.48	0.37–0.58	<2e-16
Lag 3 days	0.32	0.22–0.43	2.62e-09
Lag 4 days	0.06	−0.05–0.16	0.308
Lag 0–2 days	0.80	0.64–0.96	<2e-16
Lag 0–4 days	0.75	0.58–0.93	<2e-16

Stratified analyses showed that the association between PM_2.5_ and ischemic stroke was not different between patients ≥65 years and <65 years old or between males and females (*P* > 0.05) (Table 4).

**Table 4:** Percentage change with 95% CI in ischemic stroke admissions associated with a 10 μg/m^3^ increase in fine particulate matter (PM_2.5_) concentration by gender and age.

	Gender			Age (year)		
	Male	Female	*P* value	<65	≥65	*P* value
Lag 0 days	0.23(0.05–0.40)	0.39(0.18–0.61)	0.257	0.38(0.16–0.59)	0.25(0.06–0.43)	0.381
Lag 1 days	0.51(0.36–0.65)	0.38(0.19–0.57)	0.293	0.52(0.34–0.70)	0.44(0.28–0.60)	0.515
Lag 2 days	0.47(0.34–0.60)	0.47(0.30–0.65)	0.999	0.48(0.32–0.64)	0.46(0.32–0.60)	0.854
Lag 3 days	0.35(0.22–0.48)	0.29(0.11–0.47)	0.596	0.32(0.16–0.48)	0.33(0.19–0.47)	0.927
Lag 4 days	0.07(−0.06–0.20)	0.03(−0.15–0.22)	0.724	0.13(−0.03–0.30)	0.002(−0.14–0.15)	0.341
Lag0–2 days	0.78(0.58–0.98)	0.72(0.46–0.97)	0.720	0.86(0.61–1.11)	0.74(0.52–0.95)	0.480
Lag0–4 days	0.76(0.54–0.97)	0.72(0.43–1.01)	0.758	0.83(0.57–1.09)	0.68(0.45–0.91)	0.397

Table 5 shows the percentage changes in ischemic stroke admissions in relation to a 10 μg/m^3^ increase in PM_2.5_, under different *df* for calendar time, temperature and relative humidity. The risk estimates were stable when *df* for calendar time was 10, while different *df* for temperature and relative humidity had little effect on the estimates, suggesting that the results on the association of PM_2.5_ concentration with ischemic stroke admissions was robust in this study.

**Table 5:** Percentage change with 95% CI in ischemic stroke admissions associated with a 10 μg/m^3^ increase in fine particulate matter (PM_2.5_) concentration on the same day, by different degree of freedom (*df*) for calendar time, temperature, and relative humidity.

Variable	df	Percentage change	95% CI	*P* value
Calendar time	8	0.20	0.06–0.33	0.00474
	9	0.20	0.06–0.33	0.00445
	10*	0.31	0.17–0.45	1.57e-05
	11	0.31	0.17–0.45	1.78e-05
	12	0.30	0.16–0.44	2.7e-05
Temperature	2	0.31	0.17–0.45	1.57e-05
	3*	0.31	0.17–0.45	1.57e-05
	4	0.38	0.23–0.52	2.1e-07
	5	0.37	0.23–0.51	3.96e-07
	6	0.37	0.22–0.51	4.55e-07
Relative humidity	2	0.31	0.17–0.45	1.57e-05
	3*	0.31	0.17–0.45	1.57e-05
	4	0.31	0.16–0.45	2.38e-05
	5	0.31	0.17–0.45	2.11e-05
	6	0.32	0.17–0.46	1.3e-05

*The *df* value used in this study model.

## Discussion

The present study provides strong evidence of the association between PM_2.5_ and ischemic stroke in Beijing. The short-term exposure to PM_2.5_ was significantly associated with the first admission for ischemic stroke accounting for temperature, relative humidity, day of week, public holiday, long-term trends and seasonality of stroke events. To the best of our knowledge, this is the first city-level investigation of the relationship between PM_2.5_ and first admission for ischemic stroke in a real and severe air pollution environment. Although the magnitude of increased risk of ischemic stroke due to PM_2.5_ exposure was relatively small, the number of ischemic stroke events attributable to PM_2.5_ may be high due to the high incidence of ischemic stroke and the fact that the overwhelming majority of the public is exposed to ambient fine particulate matter, suggesting potentially large public health implications.

There has been numerous epidemiological and experiments research suggesting a possible link between air pollution and higher risk of ischemic stroke^26^. Some pathophysiological hypotheses have been proposed to explain the possible association between short-term effects of air pollution and ischemic stroke. Researchers have suggested that exposure to air pollution had adverse effects on vascular endothelial function, increased activity of the sympathetic nervous system and systemic inflammation, resulting in vasoconstriction, increased plasma viscosity, and increased risk of blood clotting and thrombosis^27–29^. These pathophysiological responses could potentially place individuals at higher risk of ischemic stroke. However, direct evidence on the association between PM_2.5_ and ischemic stroke was limited and the findings remain equivocal. A comprehensive review identified only six studies published before January 2014 that specifically examined the association between short-term exposure to PM_2.5_ and the risk of ischemic stroke hospitalization, and the pooled results suggested a non-significant increased risk of hospital admission for ischemic stroke per 10 μg/m^3^ increase in PM_2.5_^30^. Wellenius *et al*. investigated association between short-term ambient air pollution and risk of ischemic stroke in 1,705 patients admitted from Boston, USA. They found that PM_2.5_ was positively associated with ischemic stroke for the <24 hours lag period^31^. However, a study conducted in Canada, including 9,202 admissions for acute ischemic stroke, found no association between short-term increases in PM_2.5_ and ischemic stroke risk^32^. The heterogeneity of results across studies may due to differences in demographic characteristics of study population, meteorological patterns, and PM_2.5_ levels.

In this study, the estimate scaled to a 10 μg/m^3^ increase in PM_2.5_ was relatively lower when compared to previous reports. A meta-analysis that combined the risk estimates derived from previous studies reported a 1.0% increase in the daily ischemic stroke admissions per 10 μg/m^3^ increase in the level of PM_2.5_^30^. The differences in the definition of health outcomes across studies may be responsible for the weaker effect of PM_2.5_ observed in this study. Considerable attention has been focused on overall hospitalizations including admissions for both first-ever and recurrent stroke in previous studies^32–34^, possibly combining effects with different sensitivities to PM_2.5_. In contrast, only the first hospital admissions for ischemic stroke were included in this study. Epidemiological evidence indicated that individuals with a history of cardiovascular disease were more susceptible to air pollution. Patients with myocardial infarction^35^, congestive heart disease^36, 37^, or chronic obstructive pulmonary disease^38^ were demonstrated to have higher risk of death on days with heavy air pollution. Stroke survivors were at higher risk of recurrent ischemic stroke in relation to increased level of PM_10_^39^. Therefore, it is reasonable to suppose that PM_2.5_ may exert greater adverse effect on recurrent stroke than first-ever stroke, which may partly explain the relatively weaker effect observed in this study. Differences in characteristics of study population and pollutant, meteorological pattern, and exposure assessment strategy are also likely to contribute to the heterogeneity across studies. Given that this is the first study to evaluate the association between PM_2.5_ and first admissions for ischemic stroke in a badly polluted environment, the findings should be interpreted with caution and future studies are warranted to confirm the current results for PM_2.5_.

Identifying the dose-response relationship for ischemic stroke in relation to PM_2.5_ concentration is of public health and regulatory interest. Nevertheless, as prior studies were primarily conducted in Western countries with slight PM_2.5_ pollution, the exposure-response relationship in a real and severe air pollution environment remains unclear. In this study, we conducted a dose-response analysis to explore the pattern and scope of the adverse response. We observed an approximately linear exposure-response relationship, which is consistent with a time-stratified case-crossover study conducted in USA that indicated a linear relationship between PM_2.5_ and risk of acute ischemic stroke^31^. It is worth noting that, in that study, even in concentration ranges well below the present US National Ambient Air Quality Standards (20 μg/m^3^), PM_2.5_ was also significantly associated with increased risk of acute ischemic stroke, which is contrast to a relatively shallower response at low concentrations in our study. A recent time–series study conducted in China evaluated the exposure-response relationship for respiratory emergency visits in relation to fine particulate air pollution^40^. The exposure–response curve was virtually flat at low levels of PM_2.5_ (<200 μg/m^3^) and became sharp at high levels, which is consistent with our results. Our findings were also supported by a study involving 369,469 ischemic heart disease cases suggesting that PM_2.5_ at levels below 75 μg/m^3^ would not significantly increase the risk of ischemic heart disease^8^. Based on these findings, we hypothesized that there might be a threshold concentration at which PM_2.5_ become harmful enough to impose an adverse impact on the development and progression of ischemic stroke. This hypothesis may be able to partly explain the non-significant association between short-term exposure to PM_2.5_ and ischemic stroke observed in other areas where the PM_2.5_ concentrations were too low to have significant effects on ischemic stroke^32, 34^. Future studies are needed to clarify this quite important issue.

Possibly because of the higher PM_2.5_ concentration in Beijing, a stronger temporal association was noted in this study when compared to prior reports of a 1–2 day lag^31, 33^. Nevertheless, it is important to note that the pollution exposure assessment was based on the date of hospital admission rather than the time of stroke onset in this study, resulting in a substantial exposure misclassification and underestimates of pollutants effects^41^. A study conducted in USA, including 1,101 hospital admissions for acute ischemic stroke, found that hospital admission occurred at a median of one calendar day after onset of symptoms, and this delay could cause an underestimation of the association between ambient particulate matter exposure and stroke^42^. Therefore, the lag effects of PM_2.5_ exposure and ischemic stroke should be interpreted cautiously.

Examining the modification effects of individual characteristics will help to identify susceptible population and develop specific interventions for subgroups. We did not observe significant effect modification of risk by age, which is in consistent with a study indicating that air pollution risk estimates did not differ substantially between patients ≤65 years and >65 years for both ischemic and hemorrhagic stroke^43^. However, the American Heart Association made an updated scientific statement on the association between particulate matter air pollution and cardiovascular disease, suggesting that the elderly are more sensitive to the elevated PM_2.5_ level; however, the evidence is limited^9^. Elderly people are very likely to spend a greater proportion of time indoors or wear a face mask outdoors when PM_2.5_ pollution is severe, thus decreasing personal exposure^44^, which may cover up the real age-specific effects of PM_2.5_. The gender subgroup analysis showed that the associations with PM_2.5_ were not different between men and women, which are in line with previous reports^43, 45^.

Compared with prior studies that focused on the association in developed countries where PM_2.5_ pollution was mild, our study was conducted in a heavily polluted city. Given the high level of pollution, we were able to evaluate the exposure-response relationship in a wide range of PM_2.5_ concentration and have an opportunity to reveal a more complete picture of the association. Additionally, we specifically used the first admissions as the health outcome, providing a novel insight into the underlying mechanism of the association.

This study was subject to certain limitations. First, the use of air pollution data deriving entirely from one single monitoring station is expected to cause exposure measurement error, leading to underestimate the pollutant effects^46^. Second, we had no access to monitoring data on other air pollutants, such as sulfur dioxide, nitrogen dioxide, carbon monoxide and ozone, thus limiting our ability to explore the independent effect of PM_2.5_. Therefore, our findings should be interpreted with caution, and additional studies are warranted to examine the independent effect of PM_2.5_ on first hospital admission for ischemic stroke. Another limitation is our inability to differentiate the ischemic stroke subtypes, because that information was not available in our database. Future studies are needed to examine whether the acute effects of PM_2.5_ differed across strata defined by ischemic stroke etiology. Finally, the retrospective data collection may bring about bias from diagnostic and coding inaccuracy. However, both ICD-10 codes and corresponding Chinese diagnoses were used to identify eligible ischemic stroke hospitalizations, which significantly reduced bias from coding inaccuracy^47, 48^. Given the robustness of the association in all lag models, stratified analyses and sensitivity analyses, and large sample size in the present study, these potential limitations are unlikely to have compromised our results.

In conclusions, our results indicated that short term elevation of PM_2.5_ was associated with increased risk of ischemic stroke among populations in Beijing. The present study contributed to the limited scientific literature about the short-term effects of particulate matter air pollution on ischemic stroke in developing countries and additional research on this topic is warranted.
